# The Multi-Challenges of the Multi-Ion-Imprinted Polymer Synthesis

**DOI:** 10.3390/polym16192804

**Published:** 2024-10-03

**Authors:** Abraham Zepeda-Navarro, José J. N. Segoviano-Garfias, Egla Yareth Bivián-Castro

**Affiliations:** 1 Centro Universitario de los Lagos, Universidad de Guadalajara, Av. Enrique Díaz de León 1144, Col. Paseos de la Montaña, Lagos de Moreno 47460, Jalisco, Mexico; abraham.zepeda4162@alumnos.udg.mx; 2 División de Ciencias de la Vida, Carr. Irapuato-Silao Km. 12.5, Ex-Hacienda El Copal, Irapuato 36821, Guanajuato, Mexico; segovi@ugto.mx

**Keywords:** polymers, template, imprinted, ions, multi-ions

## Abstract

Multi-ion-imprinted polymers (MIIPs) are materials with a wide range of applications mainly focused on environmental recovery, mining, technology, sensors, etc. MIIPs can incorporate ions such as heavy metals, transition metals, rare earth elements, radionuclides, and other types of ions. The chemical structures of MIIPs can be designed for different purposes and with certain morphologies, such as gels, crystals, or powders, and the surface area and porosity are also considered. All these properties provide the material with several desirable characteristics, like high selectivity, high specificity, adequate efficiency, good stability, the possibility of reusability, and strategy technology adaptation. In this review, we show the multitude of challenges of multi-ion imprinted polymer chemical synthesis based on the different and interesting methods reported previously.

## 1. Introduction 

The growing demand for a discerning system capable of adsorbing diverse contaminants has propelled the advancement of novel methodologies for the synthesis of extractive and adsorbent materials. In particular, polymers have emerged as the preeminent adsorbents of choice, which is attributable to their facile synthesis, adaptable modification strategies, and formation of microporous and mesoporous structures, as well as the fact that they exhibit a higher surface area compared to other adsorbents. The polymers have emerged as a promising route for tailoring new challenges [1,2]. As a culmination of all adsorption strategies in polymers, efficient techniques such as “Molecular Imprinting Technology (MIT)” have emerged due to their easy synthesis, low cost, reusability, and greater stability. Molecularly imprinted polymers (MIPs) are designed to create artificial recognition sites within polymeric matrices, enabling them to adsorb molecules with matching size, shape, and spatial arrangement. In addition, various pores and a high surface area are created in the polymeric matrices. These structures facilitate different types of interactions between the polymer and the adsorbed molecules, including ionic interactions, hydrogen bonding, π–π interactions, van der Waals forces, and hydrophobic interactions. These interactions enhance the polymer’s adsorption capacity. Ion-imprinted polymers (IIPs) are MIPs that specifically use one type of ion as a template and have also been developed for the preconcentration or determination of ions. IIPs have been widely used in many fields, such as the identification of metal ions, environmental pollution, and the preconcentration and separation of metal ions [3,4]. In general, the synthesis process of IIPs consists of the following steps: pre-polymerization (where a pre-complex formed from the ion and monomer acts as a functional component to create the polymer matrix, along with a solvent), polymerization, and template ion elution. After the template ions are eluted, the recognition cavity complementary to the template ion is formed in the polymer matrix [5].

**Figure 1:**
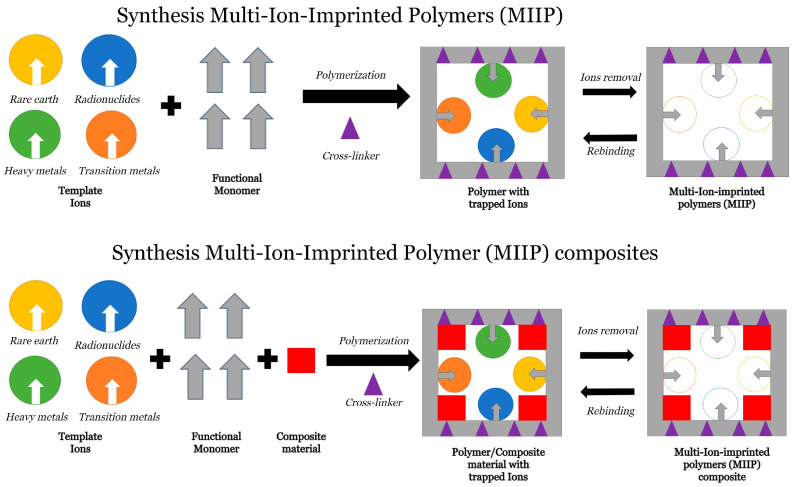
Schematic process of MIIP and MIIP composite synthesis.

In their early stages, IIPs were limited in their ability to recognize only a single target using a template ion, which limited their practical application for the removal of multiple targets. Consequently, the strategy of multi-ion imprinted polymers (MIIPs) was developed, allowing for the incorporation of multiple recognition sites within a single polymer matrix. The MIIPs and IIPs incorporate ions such as heavy metals, transition metals, rare earths, radionuclides, and other types of ions. Figure 1 shows the schematic process for a MIIP and MIIP composite synthesis.

The use of multiple ions as templates in MIIP synthesis offers a more efficient alternative compared to the preparation of separate IIPs for individual ions [5,6]. This eliminates the need for sequential preprocessing of each ion, thereby reducing the likelihood of errors. The advantages of this approach include simultaneous extraction of target ions in a single step, reduced solvent consumption (in line with green chemistry principles), cost-effectiveness, and shorter analysis times. MIIPs offer a viable and efficient solution in systems containing different competing ions, in addition to the possibility of further developing this technique with other different materials of adsorption as MIIP composites that adapt to the many challenges of pollution scenarios [7]. In this review, the possibilities offered by the multi-ion-imprinted synthesis method are presented, adapting to different scenarios that may arise.

## 2. Multi-Ion-Imprinted Polymers Utilized in Multiple Scenarios

Several metal ions are used in agriculture, technology, energy, and mining processes [8]. Therefore, the approach of recovering/detecting/removing metals by ion imprinting can be applied to various scenarios: Heavy metal ions are targeted for removal as they are well-known inorganic pollutants, primarily due to their toxicity; persistence in the environment; bioaccumulation; and higher atomic numbers, weights, and densities [9,10]. They represent a critical contaminant in water and earth because they enter human bodies via food, water, and inhalation [11]. Examples of heavy metal ions used in the synthesis of IIPs include Pb(II) [12], Cd(II) [13], and Hg(II) [12,14]. In the case of MIIPs, examples include Cd(II)-Pb(II) [15] and Hg(II)-Cu(II)-Ni(II)-Cd(II) [16,17]. Transition metal ions are extensively used for detection and recovery because of their considerable commercial significance. These metals, which include elements such as iron, copper, and nickel, play crucial roles in various industrial processes and applications. Their detection and recovery are vital for optimizing industrial processes, environmental monitoring, and resource management [18]. Examples of transition metal ions used in the synthesis of IIPs are Zn [19], Cr [20], and Cu [21]. For MIIPs, examples include Cu(II)-Ni(II) [22]. Rare earth ions are extensively utilized in diverse fields, including as functional materials and catalysts and in the semiconductor industry. Industrial applications often require pure rare earth ions, yet separating lanthanides poses a significant challenge due to their closely similar ionic radii, complicating the separation process. The challenge of recovering rare earth ions and their compounds has garnered considerable attention due to the increasing demand for high-purity materials. Rare earth elements, such as lanthanum, cerium, and neodymium, are crucial for various advanced technologies, including electronics, renewable energy systems, and high-performance magnets [18,23]. Examples of rare earth ions used in the synthesis of IIPs include Cs [24], Sm [25], and La [26]. In MIIPs, examples are La(III)-Ce(III)-Sm(III) [4] and Ce(IV)-Gd(III) [27]. Radionuclide ions appear in the environment as a result of leaching from natural deposits, release from mill tailings, emanations from the nuclear industry, combustion of coal and other fossil fuels, and release of radioactive wastewater from nuclear research. They have been used for the safe removal of ions due to their key role in ensuring the sustainable operation of nuclear power. The safe management and efficient handling of waste generated during the processing and reprocessing of spent fuel are critical aspects of this operation [28,29]. Examples of radionuclide ions used in the synthesis of IIPs are U(VI) [30] and Th(IV) [31]. For MIIPs, examples include U(VI)-Sr(II) [32]. Other, less common ions that can also be quantified and detected using IIPs include anions, nutrient ions, noble metals, and alkali metals. Examples of such ions are NO_3_^−^ [33], SCN^−^ [34], K^+^ [35], Ca(II) [36], Au(III) [37], Pt(IV) [38], Li^+^ [39], and Sr(II) [40]. One of the key factors in evaluating the selective efficiency of MIIPs and IIPs for the recovery, detection, and removal of metals is their maximum adsorption capacity. This capacity can be determined by optimizing various adsorption conditions, such as pH, temperature, adsorption time, and equilibrium ion concentrations, to identify the maximum number of ions that can be adsorbed per gram of adsorbent [10]. The following Table 1 provides an overview of research on MIIPs, including various scenarios with different mixed types of ions. It details the synthesis and polymerization methodologies used, as well as the maximum adsorption capacities of these materials.

The options for designing and selecting the types of ions in IIPs are vast and depend on the specific problems and scenarios in which each one is developed. In the recovery and detection of metals, the challenges of accurately detecting target ions are compounded by their low concentrations, potential cross-reactions with multiple ions, and interference from matrix components. Hence, there is a need to create MIIPs that possess multiple selective recognition capabilities for the purpose of extracting analytes, purifying samples, and concentrating them prior to analysis. Currently, there is a scarcity of research on multi-element analysis employing imprinted polymers. The existing studies predominantly focus on dual-ion imprinting, with limited exploration of adsorbents involving more than two types of ion imprinting [50].

## 3. Classification, Mechanisms, and Synthesis of MIIPs

The classification of MIIPs and their selective recognition abilities is primarily based on the type of interactions between the monomer and the target ion during synthesis. These interactions can be categorized into two main types: metal coordination and non-covalent interactions [51]. In metal coordination, coordination bonds are formed between the functional groups of the monomers and metal ions. The monomers used in this process contain specific functional groups that can create coordination complexes with the metal ions. In this case, the metal ions form coordination bonds with donor groups such as nitrogen, oxygen, or sulfur [52]. For example, Ali et al. utilized the functional groups of chitosan, specifically the donor nitrogen and oxygen, to coordinate the metals La(III), Ce(III), and Sm(III) to MIIP [4]. The non-covalent interactions can use ionic bonds in which ions interact with charged groups in the monomer or polymer. In this context, anionic ions can associate with cationic sites, forming electrostatic bonds between opposite charges. For example, Fu et al. utilized the functional groups of dithizone, specifically the nitrogen and sulfur anions, to facilitate electrostatic interactions with Cd(II) and Ni(II) in the synthesis of MIIPs [16]. In addition, van der Waals interactions and hydrogen bridges also play a role in this mechanism with polymers. These interactions include a variety of weak forces, such as London dispersion forces and dipole–dipole forces. Although these forces are generally weaker than other bonds, they contribute to the overall binding of the ion–polymer complex [51,53]. For example, Ali et al. and Fu et al. utilized polyvinylpyrrolidone and tetraethyl orthosilicate to create hydrogen bonds throughout the MIIP [4,16]. The interactions of MIIP in the ion absorption process may be different from the interactions in the synthesis process. In the adsorption process, MIIP may use different mechanisms.

The mechanism of MIIPs adsorption of metal ions is not unique and can be divided into two categories: physical adsorption and chemical adsorption. Chemical adsorption mainly includes complexation, chelation, and electrostatic attraction. Complexation and chelation are mechanisms that use recognition of sites by functional groups. The same size, shape, and spatial arrangement of the sites and pores in the MIIPs correspond to the target ion. These sites facilitate the binding of target ions by providing the specific characteristics necessary for effective binding to the ions. For example, Kasiri et al. describe that the adsorption mechanism for their MIIP, tetraion, involves surface complexation and chelation [17]. Electrostatic attraction, in which ions interact with charged groups, forming an electrostatic attraction between opposite charges, can be used to reduce ions. Redox is the reduction of toxic heavy metal ions to less toxic or non-toxic ions by changing their valence states [51,54]. For example, Yin et al. describe electrostatic attraction in the adsorption process of U(VI) and Sr(II) metals [32].

Physical adsorption primarily relies on weak intermolecular forces, such as van der Waals forces, rather than hydrogen bonding. While surface area does play a role in adsorption capacity, it is the nature of these weak interactions that primarily governs the process. Large surface areas are usually expected to improve the properties of solid sorbents interacting with a liquid phase by facilitating the mass transfer process and increasing the accessibility of binding sites. This type of adsorption can be in the form of multiple layers of the adsorbent material on the surface of the adsorbent material when suitable conditions of pressure and temperature are available [51,52]. For example, Xie et al. describe large surface areas as a key factor in the adsorption process of the MIIP [15].

MIIPs requires a template because the selectivity of MIIPs depends on recognition sites of the template. However, using the target ion as a template ion in traditional IIP preparation can be an environmentally harmful and costly process [55]. For example, performing the synthesis with toxic ions such as heavy metals can worsen the environmental situation due to ion contamination, and using rare earth ions can add high costs to the synthesis. For this reason, the use of non-toxic target ions as templates in the synthesis of IIP and MIIP “dummy templates” can be a green printing strategy for recognition. The dummy template will generate a binding site that is specific enough for the target compound through covalent or non-covalent bonds, and the presence of the same functional groups among the target compound and the dummy will result in an interaction between the target compound and the binding site generated by the dummy template. In recent years, the dummy imprinting strategy has been increasingly applied by using analogous ions of the target as template ions, which offers an attractive alternative [56,57]. The choice of ion will also depend on the polymerization synthesis. 

The polymerization synthesis of MIIPs can be broadly divided into three stages: (1) the selection of suitable template ions and functional monomers; (2) the polymerization of these monomers around the template ions using an initiation method such as chemical initiators, light (photopolymerization), or heat (thermal polymerization); and (3) the elution of the template ions, leaving behind “binding sites” within the polymer matrix that are complementary in size and shape to the target ion [51].The method of initiation plays a critical role in the polymerization process, with each approach offering distinct advantages and limitations. Chemical initiators are versatile and effective under mild conditions, making them widely applicable; however, residual initiator compounds may remain in the final polymer, potentially affecting its purity. Photopolymerization enables spatial control and can be initiated at ambient temperatures, which is advantageous for systems sensitive to heat, though it requires specialized equipment and is constrained by the limited penetration depth of light. Thermal polymerization is a simple and cost-effective method, but it often necessitates elevated temperatures that can degrade thermally sensitive monomers or template ions. After initiation, strategies for the synthesis of MIIPs include: (i) crosslinking linear polymers containing metal-binding ligands using bifunctional reagents; (ii) copolymerization of metal complexes with polymerizable ligands and a cross-linker; and (iii) surface imprinting at the interface of water-in-oil emulsions using amphiphilic functional monomers. Various polymerization methods can be employed in MIIP synthesis, including sol–gel, bulk, precipitation, surface imprinting, ultrasonic, thermal polymerization, self-polymerization, and emulsion techniques. The choice of polymerization method is typically determined by the desired structural properties of the polymer and the experimental conditions. These methods, combined with different initiation techniques, are integral to the successful synthesis of MIIPs [58].

Sol–gel is a process in which a metal oxide precursor is dissolved in a low-molecular weight-solvent catalyzed by substances such as acids, bases, or ions. This method can produce stable and uniform MIIPs and can form imprinted selective cavities characterized by enhanced durability and stability due to their strong and stable structures, high porosity, and voluminous nature. Template ions are incorporated into the polymer, and the sol–gel-synthesized MIIPs can have different shapes, sizes, specific functionalities, and affinities [59,60]. Research involving the synthesis of MIIPs with more than two ions uses the sol–gel polymerization method because the process is straightforward, and functional monomers in this process, such as acrylic and oxysilane, facilitate the binding of ions through coordination bonds. For example, Fu et al. first used synthesis sol–gel with 3-aminopropyltriethoxysilane as the functional monomer; tetraethoxysilicane as the cross-linker; dithizone as the chelator; and Hg(II), Ni(II), Cu(II), and Cd(II) as templates to insert into the multi-ion-imprinting adsorbent [16].

Bulk polymerization is a method where template ions, functional monomers, cross-linking agents, and initiators are dissolved in a suitable solvent. This process involves first forming a pre-polymerization complex between the template and the functional monomer, followed by the addition of a cross-linker and initiator to the solvent. After deoxygenation, polymerization is initiated either thermally or photochemically. The resulting polymer is then ground and sieved to achieve the desired particle size. The imprinted materials produced by this method exhibit high recognition sites for the target ions, as the recognition sites are uniformly distributed within the polymer matrix due to the pre-complexation step used during polymerization. However, this method requires a large amount of template, which can be economically inefficient for expensive ionic templates. Additionally, the grinding, crushing, and sieving processes used to obtain MIIP particles of specific sizes can damage binding sites, leading to reduced yield and irregularly shaped particles [58,60]. For example, Stevens et al. prepared an MIIP, triion, using the thermal bulk polymerization method. Methacrylic acid served as the functional monomer, ethylene glycol dimethacrylate was used as the crosslinking agent, and azobisisobutyronitrile acted as the initiator. Lead (Pb), zinc (Zn), and mercury (Hg) ions were used as template ions, while 1,10-phenanthroline served as the complexing agent. This MIIP showed good selectivity towards the targeted metal ion by removing 90–98% of the templated ions as compared to 58–62% of the competitive ions [41].

Precipitation polymerization is a one-step method used to produce micro- or sub-micrometer-sized polymeric beads under controlled conditions. The process starts with a dilute, homogeneous monomer solution, from which polymer beads grow and precipitate due to their limited solubility in the solvent. This method is particularly useful for synthesizing MIIPs with specific surface areas. However, it has notable drawbacks, including the extensive use of porogens during polymerization and the substantial amount of template required for synthesis. Despite these limitations, precipitation polymerization remains a popular choice for addressing particle shape irregularities that may arise from bulk polymerization [60,61]. For example, Fattahi et al. prepared an MIIP, diion, by means of precipitation polymerization for the selective extraction of cadmium and lead ions, and the results indicated that the as-prepared material had a hierarchical mesoporous structure with a high specific surface area and high adsorption capacity [46].

Surface imprinting is a method that occurs through a co-polymerization process on the surface of carriers, with most binding sites situated on the polymer surface. In this process, polymerization takes place on the surface of a solid substrate, such as silica, chitosan, or magnetite, in the presence of initiators and cross-linking agents, resulting in the formation of an imprinted layer. By removing the ion templates from the polymeric layer, three-dimensional cavities are created on the surface of the solid substrate. This approach offers numerous advantages: it is easy to prepare and widely applicable, allows for size and shape control, provides a large surface area with highly selective binding sites, and is reproducible and sensitive compared to other methods. It enhances mass transfer, ensuring complete template removal and facilitating excellent accessibility to target ions [60,61]. For example, Ghorbani et al. prepared an MIIP, diion, by surface imprinting using graphene oxide as a substrate matrix, acrylamide as a monomer, ethylene glycol dimethylacrylate as a cross-linker, and Cd(II) and Ni(II) as templates [43].

Ultrasound is a technique that can act as an initiator by breaking molecular chemical bonds and accelerating the polymerization rate, introducing a unique mechanism in ultrasound-assisted radical polymerization. Unlike conventional methods, this technique includes an additional step where radicals are generated from the cleavage of monomers and polymers. Ultrasonic waves utilize cavitation and acoustic shock effects to induce micro-streaming and micro-turbulence, which significantly enhance mass transfer rates, start-up speed, mixing efficiency, and micro-mixing. These effects improve preconcentration efficiency by reducing resistance to ion mass transfer. Additionally, ultrasonic and microwave methods offer advantages such as rapid polymerization, controlled polymer size, simple conditions, and high yield [60,62]. For example, Abdullah et al. synthesized the amine-functionalized dual-metal imprinted polymer via a simple ultrasonic-mediated precipitation polymerization approach and was applied as a magnetic adsorbent for the selective removal of Pb^2+^ and Cd^2+^ from real water samples. The application of ultrasonic heating reduced reaction time by five times as compared to conventional heating [45]. Thermal polymerization is a method that involves exposing the mixture to prolonged thermal energy, which increases the internal energy of the system and influences the formation of the monomer–template complex during the self-assembly phase. This technique often results in significantly elevated temperatures within the reaction vessel, which can lead to heterogeneity in binding sites due to increased conformational entropy. Additionally, thermal polymerization is time-consuming, typically requiring around 24 h for the synthesis of molecularly imprinted polymers (MIIPs). The high temperatures used, generally around 60 °C, may also limit the use of thermosensitive templates [63,64]. For example, Prasad et al. prepared an MIIP, diion, via thermal polymerization at 60 °C for 3 h in the presence of the template ions Ce(IV) and Gd(III), the cross-linker ethylene glycol dimethacrylate, the initiator AIBN, and multiwalled carbon nanotubes [27]. Self-polymerization is a method in which monomers polymerize spontaneously without the need for an external catalyst or initiator. The polymerization is driven by the intrinsic reactivity of the monomers, which can react with one another to form polymer chains on their own. This process is common in systems where monomers possess inherent reactivity, such as in certain chemical or biological contexts. Self-polymerization typically requires only soluble oxygen as an oxidant and does not involve complex instrumentation or harsh reaction conditions [63,65]. For example, Abdolmohammad et al. prepared an MIIP, diion, by self-polymerization using monomer dopamine, forming polydopamine for selective extraction and preconcentration of low levels of Cd(II) and Ni(II) [49]. Emulsion polymerization is a biphasic method in which hydrophobic monomers are dispersed in water using an oil-in-water emulsifier, with water acting as the continuous phase. In this process, initiators in the aqueous phase generate free radicals that start the formation and growth of polymer cores at the oil–water interface. Stabilizing agents are used to prevent the agglomeration of nucleating particles, which requires subsequent purification steps to remove surfactants from the polymer particles. While this method is effective for synthesizing polymers such as ABS, polystyrene, and PMMA, it presents challenges due to its demanding conditions and the potential impact of water on the desired hydrogen bonding interactions between polymers and templates during synthesis [60,66]. For example, Türkmen et al. prepared an MIIP, diion, by emulsion polymerization, adding the emulsified mixture to the Fe_3_O_4_-containing phase and then adding the initiators ammonium per sulfate and NaHSO_3_. These initiators initiated polymerization of the monomers in the emulsion, which occurred at 40 °C for 24 h [42]. Challenges in MIIPs and IIPs include the use of methods such as emulsion polymerization, bulk polymerization, suspension polymerization, and precipitation polymerization. Unfortunately, these techniques often result in imprinted materials with poor site recognition for target ions. This issue arises because the binding sites are deeply embedded within the polymer matrix, which negatively impacts the kinetics of the sorption–desorption process. To address these challenges, surface imprinting has been proposed as a promising solution [52]. Furthermore, the synthesis of MIIPs can be enhanced by incorporating additional techniques to optimize recovery, detection, and removal conditions. This versatility enables the creation of MIIP composites tailored to specific requirements. These composites often combine MIIPs with other compounds selected for their desirable additional properties, such as magnetic attributes, electrical conductivity, fluorescence, mechanical stability, or enhanced adsorption capabilities [63,67,68].

## 4. Multi-Ion-Imprinted Composites, Synthesis, Materials and Applications

MIIPs and IIPs have been applied for the determination, speciation, enrichment, and adsorption of trace metals; they have attracted attention due to their advantages of simplicity, low cost, reusability, and high adsorption capacity. But the same can be applied in promising areas such as sensors. Sensors are devices used to detect and respond to electrical or optical signals. MIIPs and IIPs create sensors which are widely used due to their advantages, such as good thermal, chemical, and mechanical properties; low cost; and easy and simple synthesis methods [58,63]. To enhance the sensitivity and selectivity of electrodes in electroanalysis, MIIPs are frequently engineered to target specific heavy metals. MIIPs function similarly to solid-phase extraction by preconcentrating the target metal ion onto the electrode, thereby amplifying stripping signals. This makes MIIPs particularly valuable as receptors for sensing applications. However, for optical sensors, the design is more complex, since most metal ions cannot be directly quantified through optical measurements. Consequently, incorporating a chromophore or fluoroionophore into the polymer matrix is necessary to generate or quench an optical signal (absorbance or fluorescence). Alternatively, fluorescent particles such as quantum dots can be used. In contrast, most metal ions are readily quantified using electrochemical detection methods like voltammetry or potentiometry, which do not require specific design criteria for IIPs in electrochemical sensors. This is especially advantageous for analyzing complex samples such as environmental or wastewater, human biological fluids, or solid samples, where analytical challenges may arise from interfering ions or matrix complexity. MIIPs offer several advances over conventional ion selective electrodes (ISEs), including improved selectivity and sensitivity, by creating highly specific molecular cavities tailored to target ions. This allows IIPs to outperform ISEs in the detection of ions that are difficult to measure with conventional methods. IIPs also offer greater versatility because they can be designed for a wide range of ions and integrated with different detection techniques. They typically exhibit higher stability and durability, making them more resilient under harsh conditions and potentially more cost-effective due to simpler manufacturing processes. In addition, IIPs can be engineered to reduce interference from other ions in complex matrices, resulting in more accurate measurements compared to ISEs [3,69,70]. Table 2 lists various MIIP and IIP composites and compares their polymerization methods, along with additional compounds selected for their desirable properties. These properties significantly influence their adsorption capacity and applications in sensing technologies.

Various materials can be incorporated into MIIPs and IIPs, including magnetic nanoparticles, silica particles, graphene oxide, multi-walled carbon nanotubes, and quantum dots, among others. The incorporation and polymerization of various materials with MIIPs and IIPs are carried out method mainly by “core-shell” surface ion imprinting, but different methods exist. In this approach, imprinting sites are positioned on the surfaces of supporting material cores or shells. These materials improve the ability to recognize target ions, reduce non-specific adsorption, increase adsorption capacity and selectivity, accelerate mass transfer rates, and improve analyte accessibility during the adsorption and elution steps [63,76,77]. Magnetic MIIP composites have garnered considerable interest due to their strong magnetism and specific recognition capabilities towards template ions. These composites typically utilize magnetic nanoparticles (Fe_3_O_4_) as a core support with MIIPs, and are chosen for their ease of synthesis, affordability, and minimal toxicity. Such pre-modification offers various advantages depending on the intended application, such as facilitating the preparation of stable ferrofluids and achieving desirable core–shell morphologies. Incorporating graphene oxide or multiwalled carbon nanotubes into these composites further enhances their adsorption capacities [63,76]. For example, Xie et al. prepared a dual-template magnetic ion-imprinted polymer of Cd^2+^ and Pb^2+^ using a surface imprinting technique combined with a sol–gel process, with papain as a functional monomer and magnetic mesoporous Fe_3_O_4_@mSiO_2_ as the substrate [15]. Graphene oxide (GO) and its derivative, graphene, are carrier materials due to their two-dimensional structure with a large specific surface area and abundant oxygenated functional groups, which allow for easy surface modification. These properties make GO and graphene ideal platforms for the preparation of GO/MIIP and graphene/MIIP composites, which exhibit fast adsorption kinetics, high binding capacity, and selectivity toward target ions [63,76]. For example, Bao et al. synthesized a dual-template imprinted nanoparticle material on a GO slice structure coated with MnFe_2_O_4_ magnetic chitosan film via the surface ion-imprinted technique for selective removal of Cd(II) and Ni(II) from aqueous solution [50]. Carbon nanotubes are solid materials for preparing MIIP composites due to their remarkable electrical conductivity, mechanical strength, chemical stability, thermal resilience, and expansive surface area. This allows for the creation of MIIP composites with enhanced surface area, facilitating improved accessibility and kinetic adsorption of template ions [63,76]. For example, Abdolmohammad et al. prepared a MIIP used coated on the surface of the magnetic graphene oxide sheet layer [49]. Quantum dots (QDs), semiconductor nanocrystals renowned for their exceptional properties such as intense photoluminescence, narrow tunable emission spectrum, broad excitation spectra, high quantum yield, and excellent light stability, as well as good biocompatibility, have recently garnered significant interest for integration with MIIPs. These nano-crystals, typically ranging from a few to tens of nanometers in diameter, emit diverse colors depending on their size and composition. The selective adsorption of analytes within MIIPs and IIPs is facilitated by chemical and physical interactions, which can enhance photo-luminescence or induce quenching properties. Consequently, when a quantum dot encounters an analyte ion capable of quenching its fluorescence, the intensity of emitted light diminishes, enabling quantitative analysis [63,76]. For example, Wang et al. IIP was synthesized with carbon quantum dots, with a quantum yield of 79%, the sensor showed strong fluorescence from CQDs and high selectivity due to the presence of Cu(II)-IIP [72].

Silicate minerals have been extensively studied as a means of removing contaminants due to their abundance, low cost, and high efficiency, especially those with well-defined pore structures such as kaolinite, montmorillonite, silica, zeolites, and other natural or modified silicate minerals. Silica also has good compatibility with MIIPs and IIPs, also can be easily synthesized at various size scales [3,76]. For example, He et al. synthesized ion-imprinted sulfonate functionalized silica gel polymer prepared with the surface imprinting technique by using nickel(II) as the template ion, grafted silica gel as the support, and 2-acrylamido-2-methyl-1-propanesulfonic acid and ethylene glycol dimethacrylate as the functional monomer and crosslinker [68]. The application of MIIPs and IIPs is promising due to the integration of various advanced materials in their design and synthesis. This technology is continually evolving and demonstrating remarkable improvements in its ability to recover, detect and remove metals and other ions. The incorporation of materials such as magnetic nanoparticles, graphene oxides, carbon nanotubes, and quantum dots has greatly enhanced the performance of MIIPs and IIPs, increasing their efficiency in specific applications. In addition, advances in synthesis techniques and the combination of different carrier materials have significantly expanded the range of applications and uses of these polymers. In particular, their potential as highly sensitive sensors is opening up new opportunities in many fields. Thus, the future of MIIPs and IIPs promises not only continued improvements in contaminant recovery and removal efficiencies, but also expansion into new application areas [77].

## 5. Conclusions

MIIPs mark a significant advancement in adsorption technology, addressing the limitations of traditional ion-imprinted polymers (IIPs) that target only a single ion. The capability of these advanced polymers to integrate multiple recognition sites into a single matrix enables the simultaneous extraction of several ions, optimizing both separation and enrichment processes in complex systems. This approach not only enhances efficiency by eliminating the need for separate adsorbents for each ion, but also reduces solvent consumption, operational costs, and analysis time. By facilitating the removal of multiple ions in one step, this multidimensional strategy tackles challenges related to ion similarity and mixture complexity, offering a solution for the recovery, detection, and removal of various metal ions, including heavy metals, transition metals, rare earth elements, and radionuclides, which are crucial due to their environmental and health impacts. The classification and selective recognition abilities of these polymers depend on the bonding types formed between the monomers and target ions during synthesis. These bonds can be metal-coordinating or non-covalent, such as ionic bonds, van der Waals interactions, and hydrogen bonds. Different synthesis methods, including sol–gel polymerization, bulk polymerization, precipitation, surface imprinting, ultrasonic, thermal, self-polymerization, and emulsion polymerization, offer specific advantages and challenges regarding stability, particle size, and adsorbent properties. Among these, sol–gel polymerization and surface imprinting stand out for producing materials with high selectivity and adsorption capacity. However, traditional methods can sometimes limit efficiency due to the complete immersion of binding sites in the polymer matrix, impacting the kinetics of the adsorption–desorption process. Surface imprinting effectively addresses these issues by providing a large surface area with highly selective binding sites. The incorporation of materials like magnetic nanoparticles, silica particles, graphene oxide, carbon nanotubes, and quantum dots has greatly expanded the applications of these advanced polymers in trace metal determination, speciation, enrichment, and adsorption. One key advantage is the creation of highly sensitive and selective sensors, particularly for electrochemical applications. These polymers can pre-concentrate metal ions onto electrodes, enhancing stripping signals and improving detection compared to conventional ion-selective electrodes. For optical sensors, the integration of chromophores or fluorophores is necessary to generate or quench optical signals such as absorbance or fluorescence. Additionally, materials like quantum dots enhance sensitivity through their photoluminescent properties, while carbon nanotubes provide exceptional electrical conductivity and high surface area. These innovations enable the development of composites with superior adsorption capabilities, stability under harsh conditions, improved detection, and optimized mass transfer rates.

## Figures and Tables

**Table 1:** List of selected multi-ion-imprinted polymers and the methods used for their synthesis.

Multi-Ions Imprinted Polymer	Templates	Maximum Adsorption Capacity (mg/g)	Type of Ions	Method of Synthesis	Ref
MIIP—tetraion	Hg(II), Cd(II), Cu(II), Ni(II)	105.34, 91.79, 75.03, 63.54	Transition/heavy metals	Sol–gel	[16]
	Sb(III), Te(IV), Pb(II), Cd(II)	57.8, 51.3, 61.9, 65.6	Heavy metals	Sol–gel	[6]
	Hg(II), Ni(II), Cu(II), Cd(II)	7.85, 3.15, 1.56, 3.72	Transition/heavy metals	Sol–gel	[17]
MIIP—triion	La(III), Ce(III), Sm(III)	39.34, 38.24, 40.51	Rare earth	Sol–gel	[4]
	Pb(II), Zn(II), Hg(II)	-	Transition/heavy metals	Bulk	[41]
MIIP—diion	Cd(II), Pb(II)	41.69, 76.39	Heavy metals	Sol–gel	[15]
	Cu(II), Ni(II)	-	Transition/heavy metals	Bulk	[22]
	As(III), As(V)	91.7, 99	Heavy metals	Emulsion	[42]
	Ni(II), Cd(II)	153.13, 188.67	Transition/heavy metals	Surface imprint	[43]
	Cd(II), Ni(II)	33.91, 39.35	Transition/heavy metals	Surface imprint	[44]
	Pb(II), Cd(II)	-	Heavy metals	Ultrasonic-mediated precipitation	[45]
	Cd(II), Pb(II)	18.18, 23.81	Heavy metals	Precipitation	[46]
	Cd(II), Cu(II)	-	Transition/heavy metals	Sol–gel	[47]
	Pb(II), Cd(II)	10.28, 10.38	Heavy metals	Ultrasonic- precipitation	[48]
	Cd(II), Ni(II)	-	Transition/heavy metals	Self-polymerization	[49]
	U(VI), Sr(II)	317, 160	Radionuclides	Self-polymerization	[32]
	Ce(IV), Gd(III)	-	Rare earth	Thermal polymerization/Surface imprinting	[27]

**Table 2:** List of chemical synthesis strategies used to functionalize the MIIPs.

Imprinted Polymer Composites	Combine Materials	Method of Synthesis	Application	Ref
MIIP	Magnetic	Sol–gel	adsorption	[15]
MIIP	Graphene oxide	Surface imprint	adsorption	[43]
IIP	Magnetic multi-walled carbon nanotubes particles	Emulsion	adsorption	[71]
IIP	Quantum dots	Surface imprinting	sensors	[72]
IIP	Silica	Surface imprinting/Ultrasonic-mediated	adsorption	[73]
MIIP	Magnetic	Thermal polymerization	sensors	[27]
MIIP	Graphene oxide/magnetic	Surface imprint	adsorption	[44]
MIIP	Carbon nanotube /Magnetic	Self-polymerization	adsorption	[49]
IIP	Graphene oxide	Surface imprint	sensors	[74]
IIP	Magnetic/Silica	Surface imprint	adsorption	[75]
